# [Corrigendum] Inhibition of mTOR promotes hyperthermia sensitivity in SMMC-7721 human hepatocellular carcinoma cell line

**DOI:** 10.3892/etm.2025.12973

**Published:** 2025-09-18

**Authors:** Qing-Liang Wang, Bo Liu, Xiao-Jie Li, Kun-Peng Hu, Kun Zhao, Xiao-Ming Ye

Exp Ther Med 11:961–968, 2016; DOI: 10.3892/etm.2016.2979

Subsequently to the publication of the above article, an interested reader drew to the authors’ attention that, concerning the scratch-wound assay experiments shown in Fig. 4, the “Blank control/0 h” and HT/0 h”, and “Blank control/24 h” and “HT/24 h”, data panels respectively contained sections of overlapping data, such that data which were intended to show the results of differently performed experiments had apparently been derived from the same original sources.

After having consulted their original data, the authors realized that this figure had inadvertently been assembled incorrectly. The revised and corrected version of Fig. 4, containing the correct data for the “HT/0 h”, and “HT/24 h” data panels, is shown below. Note that the errors made in assembling this figure did not affect the overall conclusions reported in the paper. All the authors agree with the publication of this corrigendum; furthermore, they apologize both to the Editor of *Experimental and Therapeutic Medicine* and to the readership for any inconvenience caused.

## Figures and Tables

**Figure 4:**
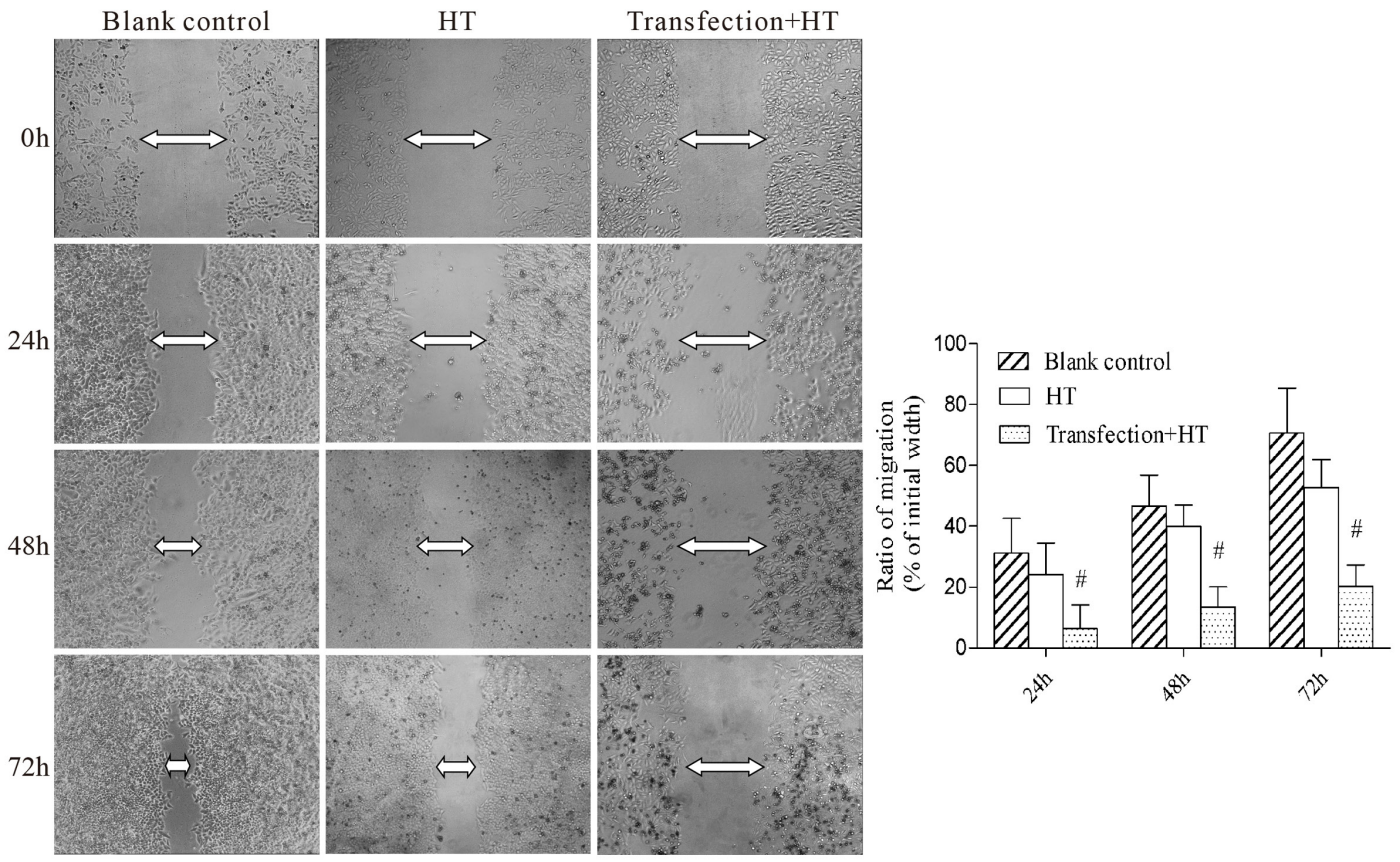
Inhibition of mammalian target of rapamycin increased the effect of HT on cell migration. Cell migration was determined by wound-healing assay. Quantification of cell migration at 24, 48 and 72 h. Mean values from three different experiments are shown. ^#^P<0.01 vs. the blank control and HT groups. HT, hyperthermia treatment.

